# Increase in Human Infections with Avian Influenza A(H7N9) Virus During the Fifth Epidemic — China, October 2016–February 2017

**DOI:** 10.15585/mmwr.mm6609e2

**Published:** 2017-03-10

**Authors:** A. Danielle Iuliano, Yunho Jang, Joyce Jones, C. Todd Davis, David E. Wentworth, Timothy M. Uyeki, Katherine Roguski, Mark G. Thompson, Larisa Gubareva, Alicia M. Fry, Erin Burns, Susan Trock, Suizan Zhou, Jacqueline M. Katz, Daniel B. Jernigan

**Affiliations:** ^1^CDC; ^2^CDC, Beijing, China.

During March 2013–February 24, 2017, annual epidemics of avian influenza A(H7N9) in China resulted in 1,258 avian influenza A(H7N9) virus infections in humans being reported to the World Health Organization (WHO) by the National Health and Family Planning Commission of China and other regional sources (*1*). During the first four epidemics, 88% of patients developed pneumonia, 68% were admitted to an intensive care unit, and 41% died (*2*). Candidate vaccine viruses (CVVs) were developed, and vaccine was manufactured based on representative viruses detected after the emergence of A(H7N9) virus in humans in 2013. During the ongoing fifth epidemic (beginning October 1, 2016),* 460 human infections with A(H7N9) virus have been reported, including 453 in mainland China, six associated with travel to mainland China from Hong Kong (four cases), Macao (one) and Taiwan (one), and one in an asymptomatic poultry worker in Macao (*1*). Although the clinical characteristics and risk factors for human infections do not appear to have changed (*2*,*3*), the reported human infections during the fifth epidemic represent a significant increase compared with the first four epidemics, which resulted in 135 (first epidemic), 320 (second), 226 (third), and 119 (fourth epidemic) human infections (*2*). Most human infections continue to result in severe respiratory illness and have been associated with poultry exposure. Although some limited human-to-human spread continues to be identified, no sustained human-to-human A(H7N9) transmission has been observed (*2*,*3*).

CDC analysis of 74 hemagglutinin (HA) gene sequences from A(H7N9) virus samples collected from infected persons or live bird market environments during the fifth epidemic, which are available in the Global Initiative on Sharing All Influenza Data (GISAID) database (*4**,**5*), indicates that A(H7N9) viruses have diverged into two distinct genetic lineages. Available fifth epidemic viruses belong to two distinct lineages, the Pearl River Delta and Yangtze River Delta lineage, and ongoing analyses have found that 69 (93%) of the 74 HA gene sequences to date have been Yangtze River Delta lineage viruses. Preliminary antigenic analysis of recent Yangtze River Delta lineage viruses isolated from infections detected in Hong Kong indicate reduced cross-reactivity with existing CVVs, whereas viruses belonging to the Pearl River Delta lineage are still well inhibited by ferret antisera raised to CVVs. These preliminary data suggest that viruses from the Yangtze River Delta lineage are antigenically distinct from earlier A(H7N9) viruses and from existing CVVs. In addition, ongoing genetic analysis of neuraminidase genes from fifth epidemic viruses indicate that approximately 7%–9% of the viruses analyzed to date have known or suspected markers for reduced susceptibility to one or more neuraminidase inhibitor antiviral medications. The neuraminidase inhibitor class of antiviral drugs is currently recommended for the treatment of human infection with A(H7N9) virus. Antiviral resistance can arise spontaneously or emerge during the course of treatment. Many of the A(H7N9) virus samples collected from human infections in China might have been collected after antiviral treatment had begun.

Although all A(H7N9) viruses characterized from the previous four epidemics have been low pathogenic avian influenza viruses, analysis of human (three) and environmental (seven) samples from the fifth epidemic demonstrate that these viruses contain a four–amino acid insertion in a host protease cleavage site in the HA protein that is characteristic of highly pathogenic avian influenza (HPAI) viruses. Chinese authorities are investigating and monitoring closely for outbreaks of HPAI A(H7N9) among poultry.

Since April 2013, the Influenza Risk Assessment Tool has been used by CDC to assess the risk posed by certain novel influenza A viruses. Although the current risk to the public’s health from A(H7N9) viruses is low, among the 12 novel influenza A viruses evaluated with this tool, A(H7N9) viruses have the highest risk score and are characterized as posing moderate–high potential pandemic risk (*6*). Experts from the World Health Organization (WHO) Global Influenza Surveillance and Response System (GISRS) met in Geneva, Switzerland, February 27–March 1, 2017, to review available epidemiologic and virologic data related to influenza A(H7N9) viruses to evaluate the need to produce additional CVVs to maximize influenza pandemic preparedness. Two additional H7N9 CVVs were recommended for development: a new CVV derived from an A/Guangdong/17SF003/2016-like virus (HPAI), which is a highly pathogenic virus from the Yangtze River Delta lineage; and a new CVV derived from A/Hunan/2650/2016-like virus, which is a low pathogenic virus also from the Yangtze River Delta lineage (*1*). At this time, CDC is preparing a CVV derived from an A/Hunan/2650/2016-like virus using reverse genetics. Further preparedness measures will be informed by ongoing analysis of genetic, antigenic, and epidemiologic data and how these data impact the risk assessment. CDC will continue to work closely with the Chinese Center for Disease Control and Prevention to support the response to this epidemic. Guidance for U.S. clinicians who might be evaluating patients with possible H7N9 virus infection and travelers to China is available online (https://www.cdc.gov/flu/avianflu/h7n9-virus.htm).

## Footnotes

* Epidemics refer to the seasonal increases in human infections; the fifth epidemic began on October 1, 2016.
